# Si-doped CsSrI_3_ perovskites as potential dielectrics in MIM capacitors: recent advances, limitations and prospects

**DOI:** 10.1039/d5ra10029a

**Published:** 2026-06-02

**Authors:** Yahaya Saadu Itas, Mayeen Uddin Khandaker, Rajesh Haldhar, Mazen R. Alrahili

**Affiliations:** a Applied Physics and Radiation Technologies Group, CCDCU, Faculty of Engineering and Technology, Sunway University Bandar Sunway Selangor 47500 Malaysia mayeenk@diu.edu.bd yitas@sazu.edu.ng; b Department of Physics, College of Science, Korea University 145 Anam-ro, Seongbuk-gu Seoul 02841 Republic of Korea; c Miyan Research Institute, International University of Business Agriculture and Technology Dhaka-1230 Bangladesh; d Department of Physics, Bauchi State University Gadau Nigeria; e NanoScience and Technology Research Group, Department of Physics, Sa'adu Zungur University Bauchi State Nigeria; f School of Chemical Engineering, Yeungnam University Gyeongsan 38541 Republic of Korea rajeshhaldhar@yu.ac.kr; g Department of Physics, School of Science, Taibah University Medina 42353 Saudi Arabia

## Abstract

Halide perovskites have emerged as the current trending materials for applications in energy storage and other high-performance optoelectronic devices due to their charge-carrier dynamics, low defect density, enhanced breakdown voltage and high dielectric profiles. Si-doped materials, such as oxides and perovskites, play a significant role in high-efficiency capacitors because of the tunable charge storage mechanisms enabled by Si. Regulating the fabrication of Si-doped CsSrI_3_ dielectric materials is a crucial step to realize a practical metal-insulator-metal (MIM) capacitor with highly sustainable characteristics. Despite their potential, a recent literature survey shows that very few works have been performed on CsSrI_3_ perovskites, prompting further research on their various properties, especially in the context of charge storage. This review highlights the recent progress in Si-doped CsSrI_3_ perovskites as dielectric chips for MIM capacitor applications. The remaining challenges in the research on Si-based CsSrI_3_ perovskite optoelectronic devices are also discussed.

## Abstract 2

Halide perovskites are promising for energy storage and optoelectronics due to low defects and high performance.

## Graphical abstract

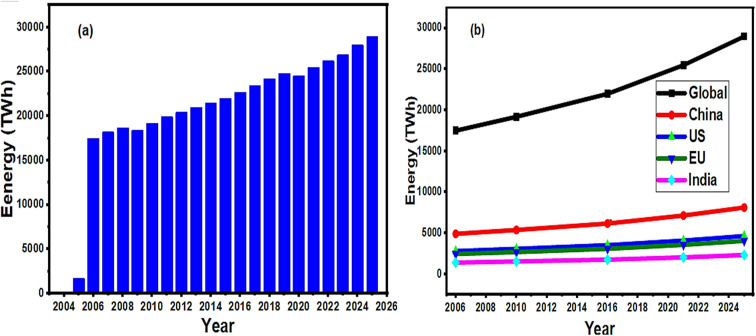

## 1 Introduction

**Fig. 1:**
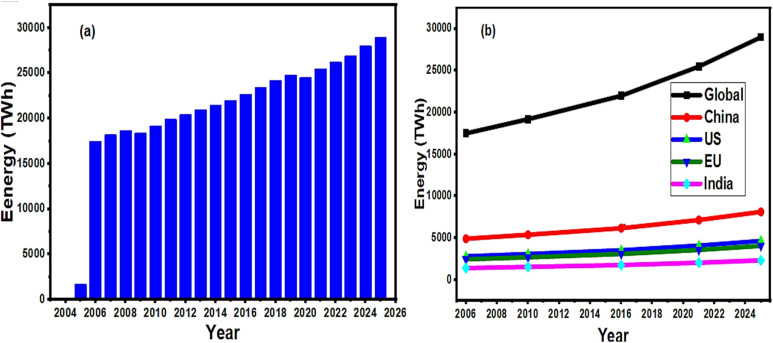
(a) Global electricity demand from 2005 to 2025 (values in TWh, based on IEA and Ember data). (b) Visualization of the global electricity demand (2005–2025) with regional breakdown and trend analysis.

A perovskite is a framework consisting of corner-sharing BX_6_ octahedra, which crystallizes based on the general formula of ABX_3_. Other perovskites with deviations from the ABX_3_ form can also be obtained when A and B cations are partially or totally vacant. Perovskites of this form are called vacancy-ordered perovskites. The most stable symmetric structure of perovskites belongs to the *Pm*3*m* cubic space group, such as SrTiO_3_. Because of the recent growth in global climate changes due to the overuse of fossil fuels, there is a need to explore better energy alternatives and robust energy storage facilities to enhance decarbonization and reduce greenhouse gas emissions.^1,2^ The most significant among these is the optimization and design of low-cost, sustainable and efficient energy storage systems. According to recent global energy reports, the current power storage capacity is limited.^3^ Projections have indicated that the year 2050 is a benchmark to achieve an increase in storage capacity by 2%.^4^ To accomplish this, it is necessary to accelerate green energy uptake and get access to advanced renewable energy systems.^5,6^ However, achieving clean, green, cost-effective, abundant and efficient energy is one of the global challenges encountered in the 21st century. Notably, solar photovoltaics, batteries, capacitors and solar thermal technologies are anticipated to offer improved solutions in the future.^7–9^ Since electrical energy demand and supply can be balanced by robust energy storage systems (ESSs), it can be generated and stored based on needs.^10^ Three main advantages of EESs include the reduced cost of supply, increased reliability, and sustained or enhanced power quality.^11^ The global energy demand has significantly overshot from 17 000 TWh in 2005 to nearly 29 000 TWh in 2025, representing a 70% increase (Fig. 1(a and b)). The only major dips identified were for 2009 (global recession) and 2020 (COVID-19). However, the post-2021 era has been witnessing significant resurgence until now. Based on the data obtained from IEA energy reviews and global electricity-demand reports, China represents 28% of global energy demand, followed by the US (16%), EU (14%) and India (8%).^12,13^

**Fig. 2:**
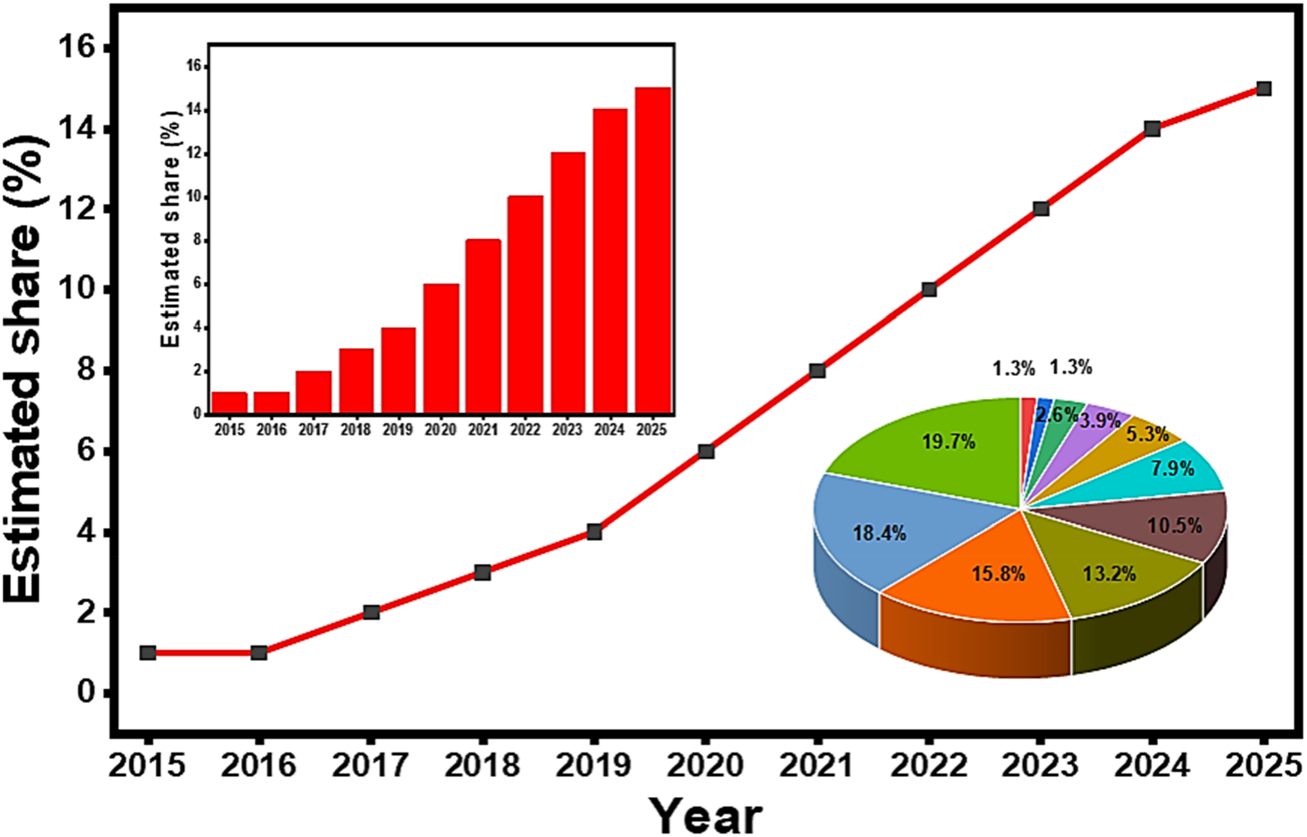
Estimated share of the perovskite use in energy storage.

In recent energy application systems, the use of fast charge/discharge capacitor materials is increasingly attracting more attention because of their outstanding potential.^14,15^ Of these charge/discharge capacitors, metal-insulator-metal (MIM) capacitors offer significant advantages over metal-insulator-semiconductor (MIS) capacitors and other capacitors because of their high energy density, low leakage current and improved frequency stability.^16^ Their mechanism depends on optimized metal–insulator–metal conditions, thereby reducing interface traps and charge accumulation characteristics of MIS designs and ensuring faster response and improved reliability.^17,18^ Lack of semiconductor components in MIM capacitors reduces parasitic effects, which allows better linearity and scalability for advanced nodes.^19^ MIM capacitors can be applied as dynamic random-access memory (DRAM), mixed-signal integrated circuits (ICs), and energy storage in various electronics.^20^ Compared to MIS, MIM capacitors provide enhanced thermal stability and integration compatibility with CMOS processes, making them ideal for high-performance systems. Despite their advantages, perovskite materials are currently neglected by the global energy markets. Direct global datasets are still emerging and are not available for reporting their percentage use. However, analytical reviews by IEA, RSC Advances, and Springer have revealed significant achievements from 2015 to date (see Fig. 2 and its insets).^21,22^ In 2015, the shares were mostly based on lab-scale studies in the context of capacitor applications. In 2020, the percentage share increased due to the use of hybrid perovskite-based electrodes in Li-ion batteries. The 15–18% projection for 2025 was based on solid-state battery and supercapacitor research; hence, there is a critical need to bring more perovskites into capacitor applications.

**Fig. 3:**
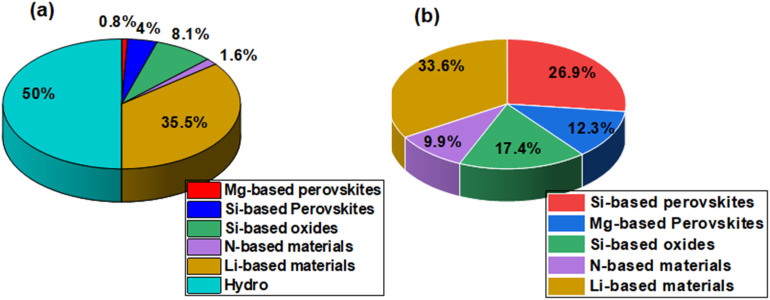
(a) Projected worldwide capacity of Si-doped MIM capacitors. (b) Percentage efficiency of Si-doped perovskites *vs.* other MIM capacitors.

According to Mukul *et al.*, doped variants of perovskites offer significant leakage reduction with enhanced long-term performance.^23^ Although they offer a high dielectric constant, better stability and better compatibility with CMOS processes, they face problems related to thermal stability, low performance due to applied stress, and limited large-scale commercialization. However, recent data, as presented in Fig. 3(a), highlights significant progress towards a global rethink on Si-doped perovskites as materials for MIM capacitors. Previous studies presented Si-doped perovskites with high energy efficiency, as shown in Fig. 3(b).^24–26^ Despite its tunable band gap (4.2 eV), cesium strontium iodide (CsSrI_3_) has been neglected in many of the optoelectronic applications.^27^ Various research works highlight the potential of CsXI_3_ (X = Sr and Sn) for use in scintillation, photodetection, and high-k dielectrics because of their reduced leakage and improved voltage breakdown strength.^28,29^ Studies indicate that CsXI_3_ are very stable to Si and Tl dopants for improved structural stability and excellent electronic properties without altering its insulating nature.^30,31^ Introducing Si enhances defect tolerance and dielectric performance, which makes CsSrI_3_ a potential material for MIM capacitors and integrated circuits. Its compatibility with CMOS processes positions it as a candidate for next-generation energy storage and optoelectronic devices. In this review, we present a comprehensive overview of the potential of CsSrI_3_ for energy storage under the influence of Si atoms for its global use in MIM capacitors. In this context, Si-doped CsSrI_3_ is a halide CsSrI_3_ perovskite optimized by substitutional doping, where Si atoms replace Sr sites. Moreover, we define Si-based perovskites as perovskite materials with enhanced electronic properties under the influence of Si impurities. Other terms used include Si-based dielectrics, Si-based capacitors, *etc.* A review of the future potential of CsSrI_3_ perovskite is necessary because only one work has been reported so far.^31^ Additionally, a review on CsSrI_3_ is significant because it will address some major knowledge gaps, consolidate scarce data, and explore its structural, dielectric, and optoelectronic potential. This foundation guides future research, material optimization, and device integration strategies.

## 2 Fundamentals of Si-doped MIM capacitors

**Fig. 4:**
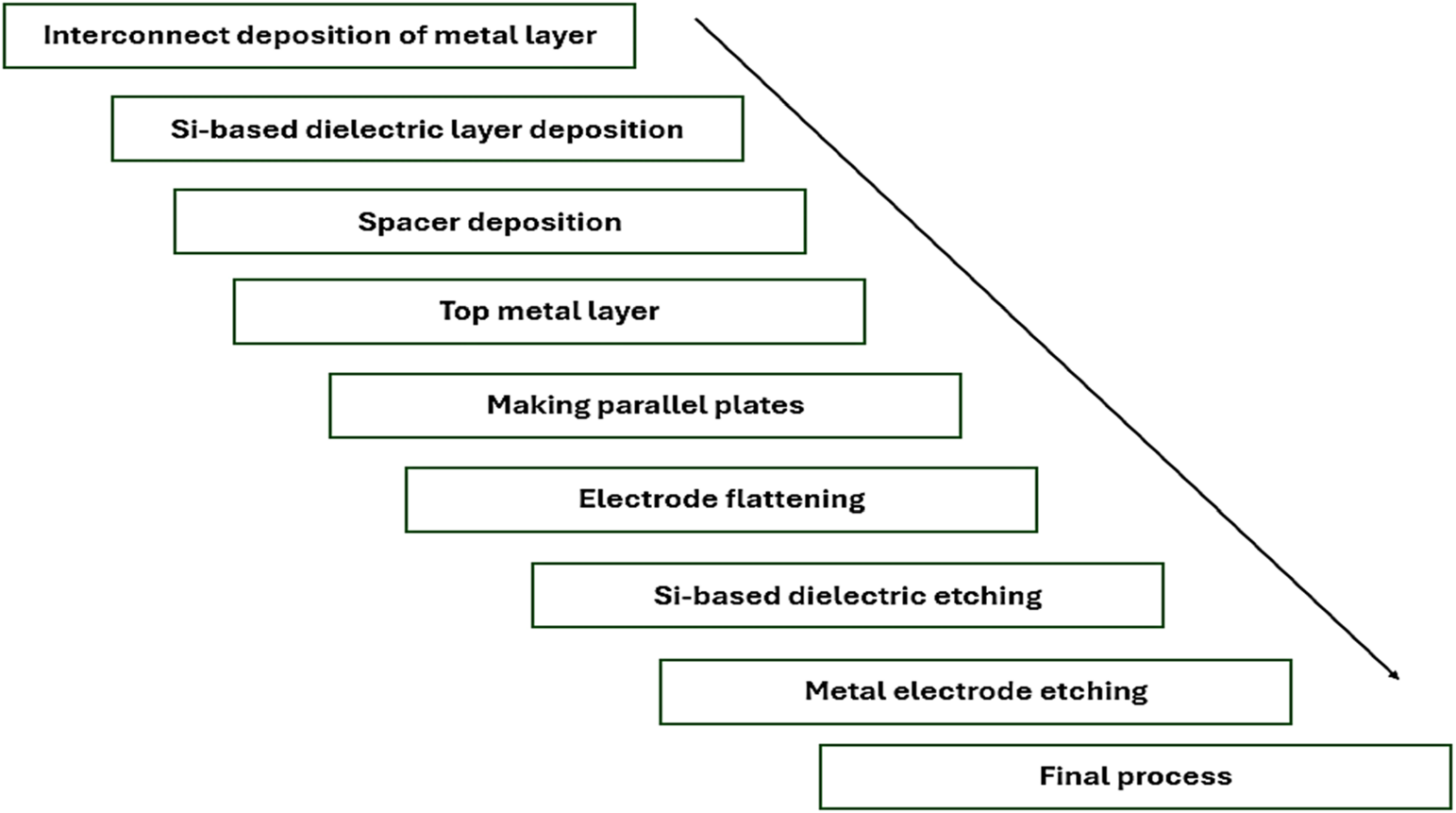
Flowchart showing the fabrication of Si-doped MIM capacitors.

Conceptually, metal-insulator-metal (MIM) capacitors refer to some high-frequency charge/discharge materials used in radiofrequency (RF) and analogue circuits because of their versatile linearity and scalability.^32^ Their structure includes an intermediate insulating component and two metal electrodes (anode and cathode).^33^ Their design is based on interface trap reduction and enhanced stability.^34,35^ On the other hand, Si-doped perovskites have been considered as promising materials for MIM capacitors due to their high dielectric constants, tunable band gaps, and high compatibility ratios.^36–38^ Their energy storage mechanisms are based on the following capacitance equation:^39^1
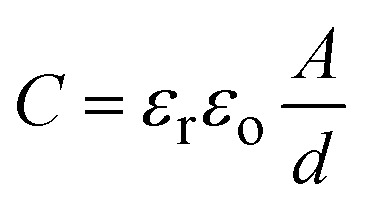
where *ε*_r_, *ε*_o_, *A*, and *d* represent the relative dielectric constant, vacuum dielectric, area of the electrodes, and dielectric thickness, respectively. We presented a fabrication process for Si-doped perovskite MIM capacitors in Fig. 4. In the initial stage, an interlayer Si-based dielectric is deposited on top of the metal layer, called the interconnect layer. The connecting metal layer is then covered with a thin Si-based dielectric layer, followed by a spacer between the dielectric layer and the connecting metal layer, which ensures a capacitor structure. To complete the process, another metal layer is deposited on top of the spacer, followed by final processing. The aim of the final process is to shield the gadget from environmental contamination; the capacitor may go through cleaning and encapsulating procedures in the final processing stage.

An extensive literature survey revealed that Si-based perovskites enhance electrical energy storage *via* strong ionic bonding coupled with a high rate of defect tolerance.^40,41^ Conventionally, this will help Si-based perovskites minimize leakage currents compared to conventional oxides. Furthermore, a wide band gap in Si-based perovskites ensures high breakdown voltage, making them suitable for miniaturized integrated circuits.^42^ In terms of dielectric capacity, Si dopants reduce oxygen vacancies and reduce structural instability, which are key sources of leakage in MIM capacitors. Based on previous studies, leakages as low as 10^−7^ A cm^−2^ and dielectric constants exceeding 100 for Si-doped perovskites have been reported, surpassing the traditional high-*k* oxides such as HfO_2_.^43^

**Fig. 5:**
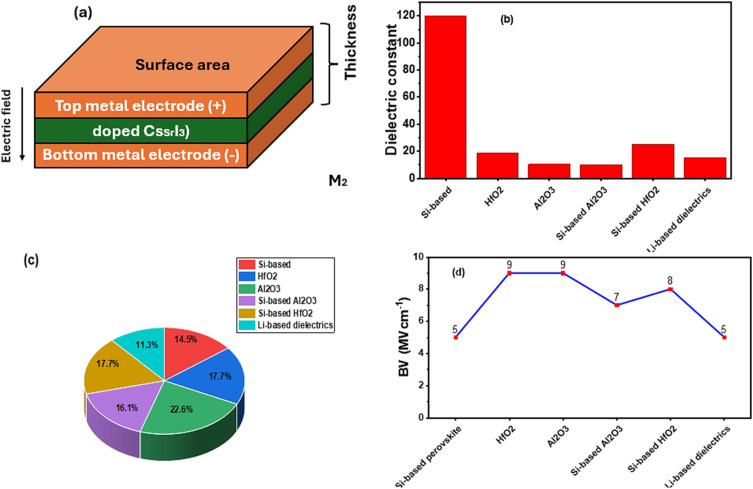
(a) Optimized form of a conventional MIM capacitor. (b) Comparison of the dielectric constants of Si-based materials with other materials. (c) Comparison of the leakage percentage of Si-based materials with other materials. (d) Comparison of the breakdown voltage (BV).

**Fig. 6:**
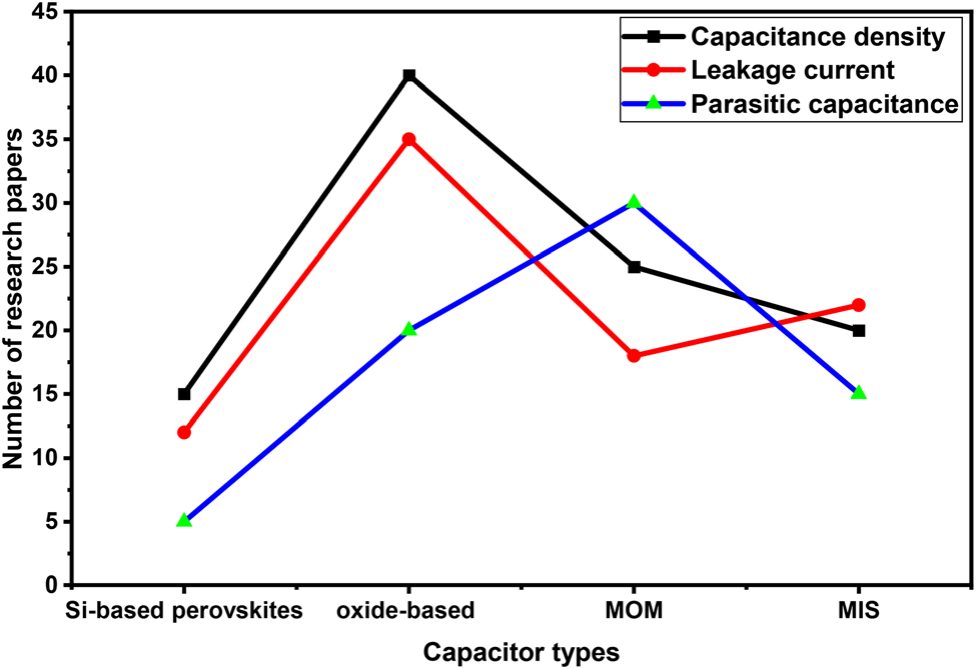
Charge storage factors *vs.* the number of research papers published.

Si incorporation improves structural stability and reduces oxygen vacancies, which are primary sources of leakage.^44^ Studies report leakage currents as low as 10^−7^ A cm^−2^ and dielectric constants exceeding 100 for Si-doped perovskites. This performance surpasses traditional high-*k* oxides like HfO_2_, offering better reliability under thermal and electrical stress. A similar work was done by Zhang *et al.*^45^ who studied Si-doped Cs-based perovskites, achieving high-*k* behaviour, better frequency response and low leakage. Other studies highlight improved CMOS compatibility and scalability for advanced nodes. These findings position Si-based perovskites as strong candidates for next-generation RF and memory applications. Despite their efficiency, some Si-based perovskites suffer from moisture sensitivity problems, thermal instability, and complex optimization processes.^45^ Therefore, the current technology requires precise stoichiometry control and defect engineering for long-term sustainability. The structural optimization of a Si-doped CsSrI_3_ MIM capacitor is shown in Fig. 5(a), while Fig. 5(b–d) present comparative charts based on conventional MIM capacitors. As shown in Fig. 5(b), Si-based materials show dominance over other reported materials, especially in terms of their superior charge storage capability, making them ideal for high-density capacitors. In Fig. 5(c), although Si-based perovskites have high dielectric constants, leakage remains a challenge compared to Al_2_O_3_, which has the lowest leakage. Fig. 5(d) shows that Si-based perovskites have lower BV than oxides, indicating a trade-off between high dielectric constant and breakdown strength. In Fig. 6, we justify the need for more research on Si-based perovskite materials in terms of energy storage.^46–50^Fig. 6 shows that research on oxide-based MIM capacitors indicates their dominance in terms of leakage and capacitance density because of their relevance to RF and DRAM applications. Research on MOM capacitors shows the major focus on parasitic capacitance due to their relevance in high frequency circuits. As observed, Si-based MIM capacitors are emerging, getting less research attention compared to other capacitors, making it essential to review their future potential in optoelectronic devices.

### 2.1 Applications of Si-doped capacitors

In Si-doped capacitors, silicon plays key roles as substrate, dopant, or dielectric modifier to improve efficiency.^51,52^ Various review and experimental works have suggested this type of capacitor for advanced electronics due to the excellent thermal, electronic and mechanical properties of silicon.^53,54^ Jia-Chuan *et al.*^55^ reported enhanced capacitance in porous Si-based materials *via* three-dimensional electrochemical etching of graphene.^55^ Their work showed improvement in specific capacitance with laser trenches, correlated with lateral etching applicable in VLSI technology. Dongxia *et al.*'s experiments revealed the activity of Si dopants in enabling a longer life in lithium-ion batteries.^56^ They found that Si-doped NCM (NCM-Si) exhibits an impressive 89.8% capacity retention rate after 300 extended cycles, while the unmodified NCM only achieves 67.0%.

**Fig. 7:**
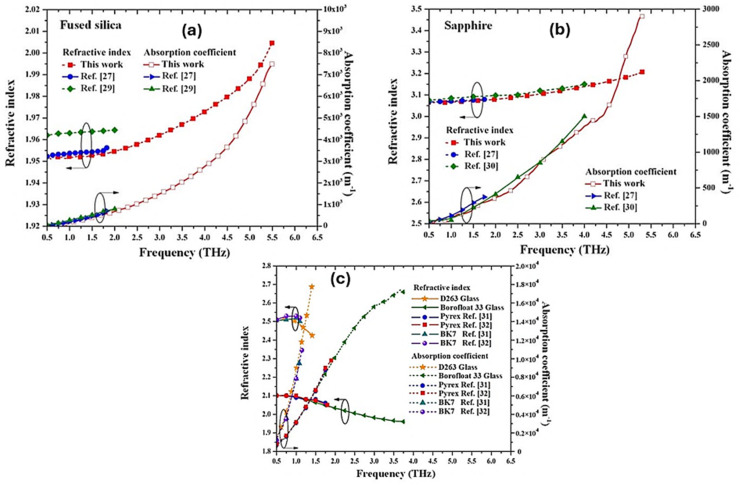
Extracted refractive indices and absorption coefficients obtained from reports by Nonchanutt *et al.*^60^ and others for (a) fused silica wafer, (b) sapphire wafer and (c) borosilicate glasses. Obtained with permission under the IOP-CC BY copyright.

**Fig. 8:**
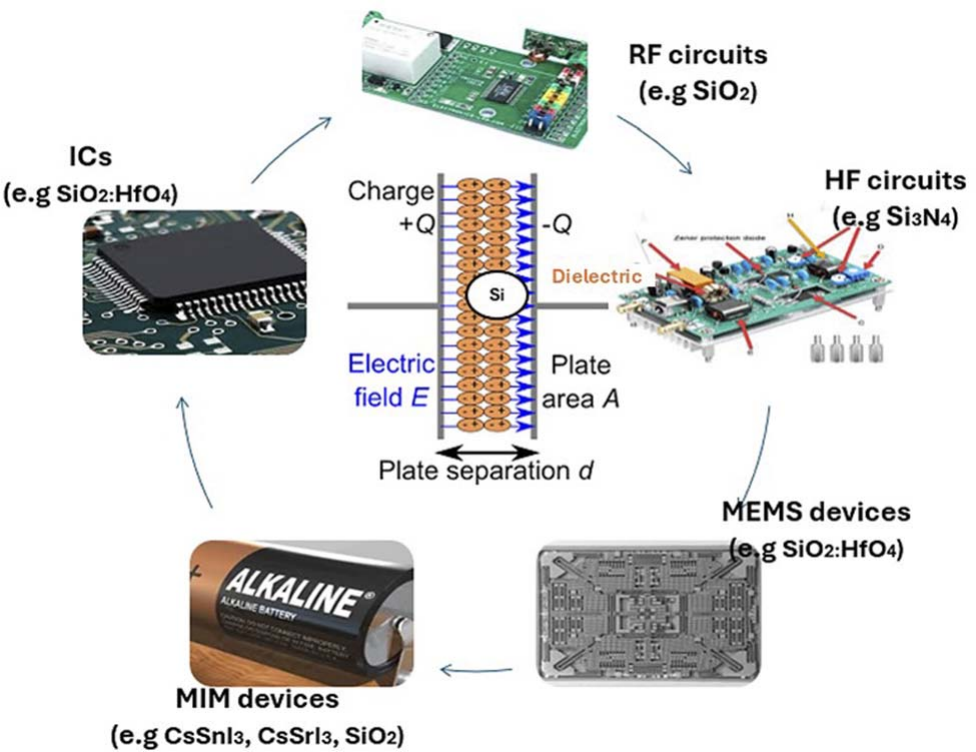
Relevance of the Si-doped dielectric materials in various semiconductor technologies.

Silicon is considered the backbone of semiconductor science and technology because it can be easily fabricated and integrated into ICs.^57–59^ In the research by Nonchanutt *et al.*,^60^ six commonly used silicon and glass substrates were used to investigate optical, electrical and dielectric properties. Refractive index, absorption coefficient, dielectric constant and loss factor were also considered. Characterizations were done *via* THz time-domain transmission and reflection spectroscopy with measurement frequencies ranging from 0.5 THz to 6.5 THz. Their results revealed that most of the selected samples are suitable for THz integrated circuits (THz ICs), THz microsystem technologies (THz MSTs), THz system-on-a-chip (THz SoC), and system-on-substrate (SiP) with minimum loss in the range of 0.042 × 10^−3^ to 0.127. Nonchanutt *et al.* compared their findings for the extracted refractive indices and absorption coefficients with previous works, as shown in Fig. 7(a–c). Fig. 7(a) compares the refractive index and absorption coefficient for fused silica and sapphire. For fused silica (Fig. 7(a)), refractive indices at 1.75 THz are 1.955, 1.964, and 1.952, respectively, with a maximum deviation of 0.012 compared to previous works. The corresponding absorption coefficients are 605 m^−1^, 685 m^−1^, and 533.18 m^−1^, all showing an increasing trend with frequency. For sapphire (Fig. 5(b)), previous data and this work extend up to 1.75 THz and 4 THz. At 1.75 THz, refractive indices are 3.079, 3.095, and 3.075, with a maximum difference of 0.65%. Absorption coefficients at this frequency are 372 m^−1^, 330 m^−1^, and 297.57 m^−1^. Data from Fig. 7(c) showed that the Si content adjusts the refractive index, stress, and dielectric loss for high efficiency. Fan *et al.* also revealed that Si-based RF capacitors (such as silicon-integrated epitaxial lead-free BaTiO_3_-based capacitors) show high-*k* for large capacitance density, stable Q, and leakage control.^61^ For example, Si_3_N_4_/SiON dielectrics have been known for efficient charge-trap engineering and reliability trade-offs.^62^ Si-doping in Ba and Sr titanate perovskites (XTiO_3_; X = Ba and Sr) improves leakage, breakdown, and field-induced stability under large RF bias swings.^63,64^ In MEM systems such as sensors, actuators and microphones, silicon-containing networks provide controllable stress, low leakage and CMOS-compatible deposition critical for MEMS reliability and yield. In terms of structural membrane and isolation performance, LPCVD Si_3_N_4_ shows high tensile stress and low permeability. It also displays chemical robustness and high breakdown, which are necessary for actuator and sensor technologies. Overall, the application of Si-doped dielectric materials shows that perovskites also actively accommodate Si dopants for power efficiency. Various applications of Si-doped perovskites in optoelectronic chips are presented in Fig. 8.

### 2.2 Si-doped perovskites as energy storage devices

**Fig. 9:**
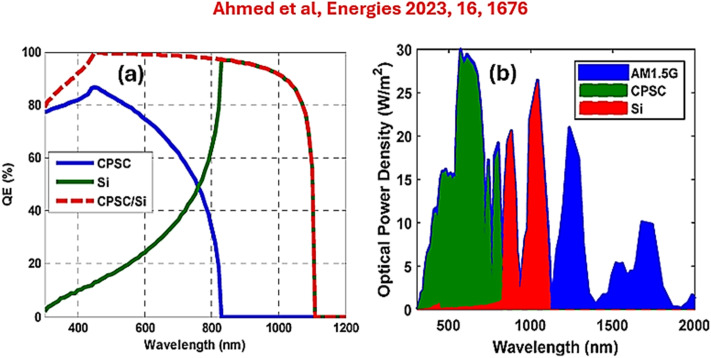
(a) EQE patterns of CPSC/Si sub-cells and tandem solar cells. (b) Absorbed AM1.5G spectrum of the CPSC/Si tandem solar cell, according to Ahmed et al., Copyright@CC BY 4.0.

Perovskites belong to the class of crystalline materials with the universal formula ABX_3_, where A and B represent cations with different sizes.^65,66^ X stands for an anion, specifically oxygen and halides such as Br and I. In the structure, the large cation (A-site) occupies the corners of the cubic unit cell, while the B-site (small cation) occupies the centre with X anions at face centres. The atomic arrangements in perovskites form a highly symmetric crystal lattice with tunable electronic and optical properties *via* chemical substitution.^67^ Silicon atoms, either as dopants or partial substituents, form an Si-doped perovskite structure with modified lattice parameters, electronic band structure and defect chemistry, which enhances the dielectric and stability characteristics.^68^ As already stated, good materials for MIM capacitors shall possess a high dielectric constant (*ε*_r_), low leakage current, and thermal stability. Conventional perovskites have a high *ε*_r_, making them attractive for energy storage. However, their performance is often limited under high electric fields and unfavourable temperature cycling.^69^ Hsing-I *et al.* investigated the dielectric properties of BaTiO_3_ and Ba_0.95_Ca_0.05_TiO_3_ sintered in a reducing atmosphere.^70^ They identified oxygen vacancies, which led to lower leakage currents at high temperatures. Other works on perovskites such as SrTiO_3_, CsSnI_3_, CsSrI_3_ have been reported and significant high dielectric constants were obtained.^71–73^ Si-doped perovskites introduce silicon either at the B-site or as a co-dopant, which can reduce oxygen vacancy concentration, improve breakdown strength and stabilize phase transitions.^74^ Together, these project a consistent dielectric response across a wide temperature range. For example, Si-doped BaTiO_3_ can maintain high permittivity while reducing dielectric loss, making it suitable for high-density capacitors in RF and power electronics.^75^ Regulated Si doping can also balance high permittivity with low dielectric loss, optimizing energy storage density and reliability. According to several reports, the incorporation of silicon into perovskites addresses key issues, resulting in improved chemical stability (*via* powerful Si–O bonds), lowered temperature (*via* thin-film deposition), enhanced compatibility, and tunable electronic properties.^76–78^ Various reports have indicated that Si-based perovskites have been widely investigated for tandem solar cell applications. Table 1 presents data on the power conversion efficiency (PCE) of Si-based perovskites (carbon-perovskite/silicon and carbon-perovskite/CIGS-GeTe tandem solar cells) in the context of tandem solar cell applications, obtained from the literature. On the other hand, Ahmed *et al.*'s work on high-efficiency and stable carbon-perovskite/silicon and carbon-perovskite/CIGS-GeTe tandem solar cells, providing significant information on Si-based perovskites.^79^ Based on the output of their work, the cell output factors of the tandem at 250 nm included a PCE of 19.89%, FF of 85.1%, *V*_OC_ of 1.66 V, and *J*_SC_ of 14.08 mA cm^−2^. The EQEs for the top, bottom, and tandem can be found in Fig. 9(a). In Fig. 9(b), the absorbed spectrum of AM1.5G by the CPSC/Si tandem solar cell was realized. The cut-off wavelength of this tandem solar cell was about 1120 nm, according to the energy gap of silicon. The results obtained by Ahmed *et al.* justified the active performance of Si-doped perovskites in various energy-capturing applications.

**Table 1:** PCE data from recent studies on tandem solar cells

PCE (%)	Dielectric	Ref.
19.89	CPS/Si	79
21.40	PSC	80
22.50	PSC/Si	81
22.70	PSC	82
23.60	PSC/SHJ	82
24.69	CPSC/CIGS-GeTe	82
25.00	Four-terminal PSC/CIGS	82
23.60	PSC/SHJ	83

### 2.3 Si-based perovskites as photovoltaics and supercapacitors

Si-based perovskites have been recently trending as promising materials for next-generation supercapacitors (SCs) because of their versatile electronic structures, high dielectric constants and flexible band gaps. Their electronic mechanisms are based on fast surface redox reactions and charge separation due to induced lattice distortion. The overall behaviours in Si-based perovskite supercapacitors enhance carrier mobility and reduce recombination losses. Jose *et al.* reported that Si-doped Sr_1−*y*_Ca_*y*_MnO_3_ exhibits improved specific capacitance and energy density compared to the conventional Sr_1−*y*_Ca_*y*_MnO_3_.^84^ The resulting enhancement depends on the synergistic effect of high surface area and optimized electronic states introduced by Si. According to Tiefeng *et al.*, the dielectric constant of Si-doped Si/SiO_2_/WS_2_/(CH_3_NH_3_)_*n*+1_Pb_*n*_I_3*n*+1_NS/Au perovskite showed a significantly higher magnitude than conventional materials, allowing for superior charge storage.^85^ On the other hand, leakage current poses a significant challenge, mostly due to defects and grain boundaries formed. With proper regulation, during synthesis, Si doping can mitigate leakage by stabilizing the crystal structure and reducing ionic migration.

Another key area that benefits from the Si-based perovskite technology is the power conversion, where various Si-based perovskites have been used in photovoltaics. A review by Xiaohui *et al.* reported that doping with organic atoms (including Si) regulates band structure and introduces shallow states, enhancing charge separation and mobility.^86^ In the context of photovoltaics, the process leads to a reduced rate of recombination losses with improved carrier mobility, leading to high power conversion efficiency (PCE). In supercapacitors, similar characteristics improve electronic conductivity and increase ion diffusion, which are necessary for achieving high power density with better charge/discharge efficiency. This review critically investigated Fangying *et al.*'s experiment^87^ on Si-based Cs_2_NaErCl_6_:Sb^3+^ electroluminescent light-emitting devices (LEDs) and obtained an additional infrared state at 1.5 µm. According to their findings, Si atoms improve crystallinity, coupled with reduced trap states, which is beneficial for PCE and leakage mitigation in dielectric-based energy storage systems. They also found that Si-dopants lowered self-discharge rates and improved the life cycle, which is good for both PVs and SCs. The interesting part of their finding is that the external quantum efficiency (EQE) of a Cs_2_NaErCl_6_:Si-based optoelectronic device increased with current density, reaching about 2% at 300 mA cm^−2^. Based on the insights gained, Si-based perovskites could be robust electrodes for hybrid energy storage systems such as MIM capacitors. Their tunable electronic properties, combined with high dielectric constants, make them promising candidates for integrating energy conversion and storage in a single platform.

### 2.4 Si-based perovskites photo-capacitors and photodetectors

Photodetectors are highly significant in military, aerospace, communications and biochemical imaging.^88^ A photodetector is a device used for identifying and measuring photon flux by converting absorbed photons into a photocurrent *via* the photoelectric effect. Photodetectors use a semiconducting material for absorbing incident photons and generate electron–hole pairs under photoexcitation. According to Anupam *et al.*, performance parameters for photodetectors include response time (rise and decay time), detectivity (D*, Jones) and external quantum efficiency (EQE, %).^89^ Others include the photodetective gain (*G*), responsivity (*R*, AW^−1^), and photocurrent/dark-current ratio. Because of the complex fabrication processes of inorganic semiconductors (*e.g.*, Si and GaN) and photodetectors, solution-processable materials, such as halide perovskites, have been considered and have shown good potential for preparing photodetectors using a simple process with a low cost and high responsivity.^90^

**Fig. 10:**
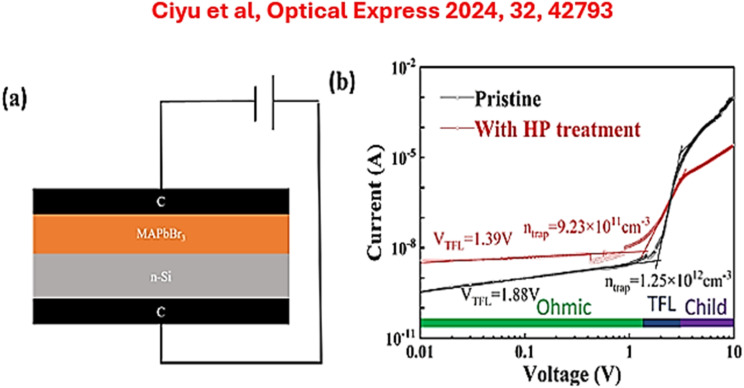
(a) Structural profile of the C/Si/MAPbR_3_ photodetector. (b) SCLC patterns of MAPbR_3_ according to Ciyu *et al.* Copyright@CC BY.

Under illumination, photons absorbed by Si-based perovskites excite electrons from the valence band to the conduction band, creating electron–hole pairs. The newly created shallow states act as charge-transfer channels and accelerate carrier mobility. With reduced trap-assisted recombination, low dark current and high signal-to-noise ratios are obtained, which are essential for sensitive photodetection. By considering the potentials of silicon-based microelectronics, Ciyu *et al.* (Fig. 10(a and b)) optimized a high-quality silicon-based perovskite photodetector with the detectivity exceeding 7.5 × 10 Jones.^91^ According to their findings, Si-based MAPbBr_3_ demonstrated photoresponsive performance with a responsivity of 11.7 A W^−1^, a detectivity of 7.57 × 10^13^ Jones, and a linear dynamic range of 147 dB at 405 nm with a 0.5 V bias. Moreover, the device can respond to light with its light intensity as low as 2.14 × 10^−6^ mW cm^−2^, demonstrating excellent weak-light detection performance.

The review by Jie *et al.* indicated major challenges regarding the operational process of Si-based perovskites, such as moisture sensitivity, phase segregation under prolonged operation, and scalability of fabrication.^92^ Yan *et al.* suggested defect passivation, interface engineering, and hybrid architectures combining Si-perovskites with transport layers or polymers to enhance durability and reduce leakage.^93^ To achieve high responsivity, low-dimensional perovskites, together with other low-dimensional materials like graphene, tungsten disulfide and molybdenum disulfide, have been combined to fabricate photodetectors with unique structures and photoelectric properties. Previous works on the performance parameters of Si-based perovskite photodetectors are listed in Table 2. As observed in Table 2, the Si-doped perovskite photodetectors show broad photoresponses in the range of 400 to 700 nm. They also show high light sensitivity with an average maximum spectral responsivity of 1.25 × 104 AW^−1^. Moreover, Yang *et al.* proposed a photodetecting system based on CsPbI_3_ nanowires and 1D all-inorganic CsPbI_3_ nanorods. Synthesized *via* a solution process, the CsPbI_3_ nanowires demonstrated a responsivity of 2.93 × 10^3^ A W^−1^ (see line 1 in Table 2). Another mixed-dimensional heterostructured Si-based perovskite photodetector demonstrated an enhanced photoresponsivity of 7.7 × 104 A W^−1^, with a rise/decay time of 0.59/0.32 ms. Yue *et al.* also fabricated an all-perovskite photodetector with high sensitivity, with an active 405 nm visible spectrum and responsivity of 1.81 × 10^3^ AW^−1^ (see line 7 of Table 2). On the other hand, Si-doped LED perovskites were also reported (see lines 8–10 of Table 2). In the context of photo-capacitance, Zeyu *et al.*^94^ reported a single perovskite that simultaneously harvests light and stores electrochemical energy. Photon absorption causes electronic excitation from the VB to the CB, prompting photogenerated electrons to migrate towards the current-collecting electrode. The positive holes move towards the opposite site, causing some portion of the charges to be electrostatically stored at the electrode–electrolyte interface. As illumination continues, carriers are injected continuously, and the capacitor is charged *via* a solar-driven process. Aleena and Tiwari investigated silicon-perovskite tandem solar cells, achieving an efficiency of approximately 34.85%.^95^ Their work primarily focused on 2T monolithic silicon–perovskite TSCs.

**Table 2:** Some previously conducted works on Si-doped perovskite photodetectors

Sl. no.	Perovskite	Optical wavelength (nm)	Rise/decay time (ms)	*R* (AW^−1^)	Ref.
1	Si/SiO_2_/CsPbI_3_	405	0.05/0.15	2.93 × 10^3^	96
2	Si/SiO_2_/CsPbBr_3_ nanoplates	442	0.6/0.9	3.4 × 10^1^	97
3	Si/SiO_2_/graphene/CsPbCl_3_ NC/Au	400	0.30/0.35	10^6^	98
4	Si/SiO_2_/CsPbBr_3_ MP/Au	442	1.8/1.0	1.8 × 10^−1^	99
5	Si/SiO_2_/CsPbI_3_ NW arrays	630	0.85/0/78	1.29 × 10^3^	100
6	Si/SiO_2_/MoS_2_/CsPbI_3−*x*_Br_*x*_ QD/Au	532	0.59/0.32	7.7 × 10^4^	101
7	Si/SiO_2_/CsPbCl_3_ NW	405	—	1.18 × 10^3^	102
8	CsPbBr_3_ QDsAl/p-Si/perovskite	512	—	—	103
9	MAPbI_3_ films Al/p-Si	785	—	—	104
10	CsPbI_3_ QDs Al/p-Si/perovskite	685	—	—	105

## 3 Si-doped CsSrI_3_ perovskites

**Fig. 11:**
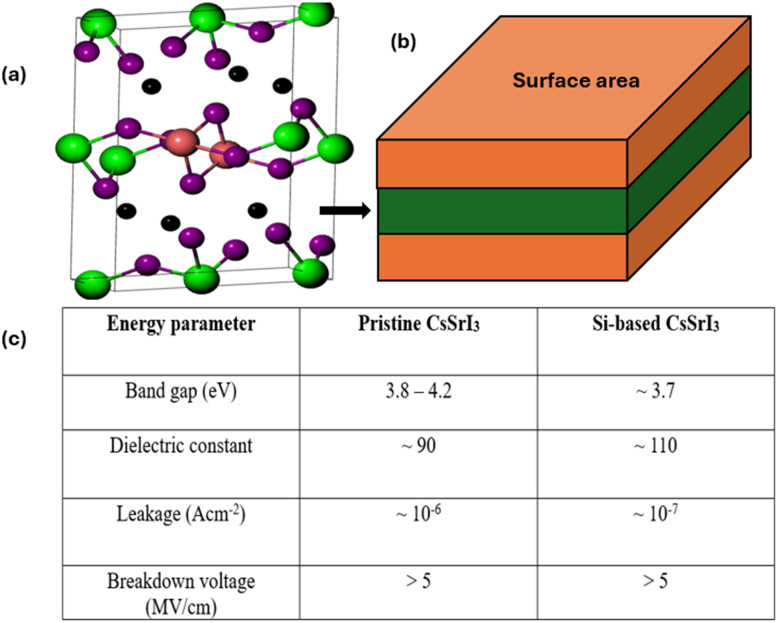
Structural and electronic properties of Si-doped CsSrI_3_ perovskites. (a) Optimized crystal structure of Si-doped CsSrI_3_ showing lattice arrangement and atomic positions, where Si substitution modifies the local bonding environment. (b) Schematic illustration of the layered configuration highlighting the effective surface area relevant for device applications. (c) Comparison of key electronic parameters between pristine and Si-doped CsSrI_3_, showing slight band gap reduction, enhanced dielectric constant, reduced leakage current and maintained high breakdown voltage.

Caesium strontium iodide (CsSrI_3_) belongs to the group of perovskites known as halide perovskites.^106^ It has a wide band gap in the range 3.8–4.2 eV, making it highly insulating and suitable for the physics of dielectric materials. The ABX_3_ structure consists of Cs^+^ at the A-site with Sr^2+^ located at the B-site. The X-site provides space for I^−^ ions. In halide perovskites, incorporating Si atoms either by doping or substitution improves their structural stability, dielectric properties, and leakage stability, making them promising for high-k MIM capacitors.^107^ Due to its excellent thermal stability and strong ionic bonding characteristics, CsSrI_3_ is highly resistant to defect formation. With Si dopants, its lattice stability can be improved with minimal vacancy defects.^108^ Based on this, there will be enhanced reliability under high electric fields. Its high chemical stability makes it compatible with CMOS processes, a critical requirement for integrated circuits. Since CsSrI_3_ has a relatively large band gap, there is a high probability of low electronic conductivity and minimum leakage. Though introducing Si may slightly modulate the band gap, the insulating behaviour can be maintained. Si modification only improves defect tolerance and decreases trap-assisted conduction, resulting in leakage currents as low as 10^−7^ A cm^−2^. Studies by Manwen *et al.* revealed that Si doping increases the dielectric constant beyond 100, which is relatively higher than conventional oxides like Al_2_O_3_ (*ε*_r_ ≈ 9) and HfO_2_ (*ε*_r_ ≈ 20–25).^109^ Previous works by Tridip *et al.* and Hang *et al.*^110^ revealed that the attributed high dielectric constant and BV of >5 MV cm^−1^ enables Si-doped ABX_3_ perovskites to store large amounts of electric charge.^110,111^ Furthermore, other works reported Si doping as the driving force for the enhanced polarization response and a capacitance density of >20 fFµm^−2^, applicable for RF and DRAM circuits. Recent studies have indicated Si-doped Cs-based perovskites in high-k applications. These works found enhanced dielectric performance, minimum leakage, and efficient reliability compared to undoped perovskites. The energy storage mechanism relies on ionic displacement within the perovskite lattice under an electric field, enhanced by Si-induced defect passivation. This mechanism ensures high polarization and minimal energy loss during charge–discharge cycles. Fig. 11(a–c) presents a summary of the structural and energy storage properties of Si-doped CsSrI_3_ perovskites.

### 3.1 Electrical and dielectric behaviour

**Fig. 12:**
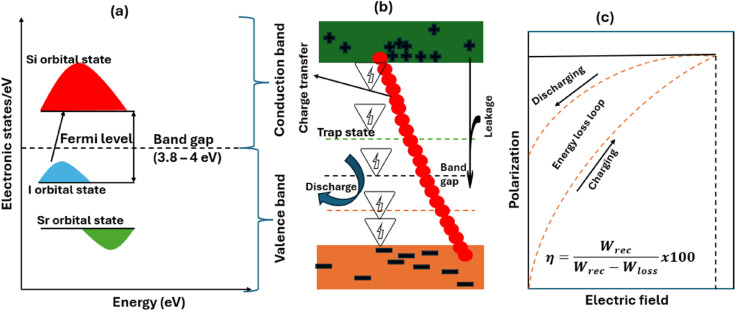
(a) Orbital states and energy level diagram of the CsSrI_3_ perovskite. (b) Energy storage activity in CsSrI_3_. (c) Charging and discharging process in CsSrI_3_.

The illustrated energy–state diagram presented in Fig. 12(a) is designed based on DFT data from various works.^112^ It reveals the distribution of electronic states across various energy levels. Mechanistically, the valence band (VB) is composed of p-orbitals for iodine and caesium, with s-orbitals occupying the lower region. On the other hand, the conduction band (CB) is primarily dominated by d-orbitals. A wide band gap can be seen in the DOS diagram, ensuring strong insulating properties, which are critical for minimizing leakage currents. The Si atoms introduced shallow energy states near the edge of the CB, called trap states (Fig. 12(b)), significantly reducing defect-related mid-gap states in Si-doped CsSrI_3_ perovskites. Therefore, this material has strong potential for improved charge transport under an external electric field without changing its insulating properties. Leakage parameters and the energy storage capacity of the CsSrI_3_ perovskite are shown in the simplified band diagram (Fig. 12(c)). Electrons are ideally stored/trapped in the state indicated by red dots. During leakage, electrons make unwanted movements towards the valence band or *via* dielectric breakdown, leading to the loss of stored charge. Energy storage is created by the applied field, causing electrons to migrate to higher energy states, known as localized traps, which are later released in a controlled manner whenever the circuit demands energy. Beihai *et al.* identified that excessive leakage in capacitors is due to trap-assisted tunnelling and dielectric breakdown.^113^ They also identified that a high electric field prompts trapped electrons to facilitate leakage behaviour. Therefore, it is necessary to carefully regulate intense electric fields, trap energy, dielectric breakdown and charge/discharge mechanisms for better energy capture.

ABX_3_ perovskites, such as CsSrI_3_, accumulate electrical energy by either dynamic random access or charge trapping. In the mechanism of dynamic random access, strong external voltage causes charge separation with excess electron accumulation around the electrodes of the capacitor. The corresponding charge deficiency is then formed on the other electrode across a dielectric material. The resulting energy is stored in the electric field established between the positive and negative electrodes due to this charge imbalance. A prominent research work reported that the energy stored based on dynamic random access slowly leaks over time. For the charge trapping aspect, ABX_3_ perovskites serve as an insulating layer coated around a conductive electrode to physically trap electrons. In this concept, high voltage forces electrons through the insulating layer to a floating gate or trap layer. These trapped electrons will remain isolated because of the effects of the ABX_3_ insulating layer coating.

Band gap regulation is the most appealing procedure for tuning the band gaps in halide perovskites for efficient energy capture. Since most of the halide perovskites typically have wide band gaps, it is necessary to bridge their performance between leakage and efficiency. Regarding this, their band gap must be adjusted to the optimal value for efficient energy storage (around 3.5 eV). Semiconducting materials with narrow band gaps are highly susceptible to excessive leakage currents. On the other hand, too large band gaps exhibit very slow energy capture. For the benefit of readers, we have identified band gap modulation strategies, such as the substitutional approach, pressure approach and reduced dimensionality approach. Evaluations of the energy storage performances of ABX_3_ materials that have the same physical and chemical properties as Si-doped CsSrI_3_ (*e.g.*, Si-doped CsSnI_3_, Si-doped CsPbI_3_ and Si-doped BaTiO_3_) have been reported. For a linear dielectric material, the energy storage density (*W*) is determined using the following equation:2
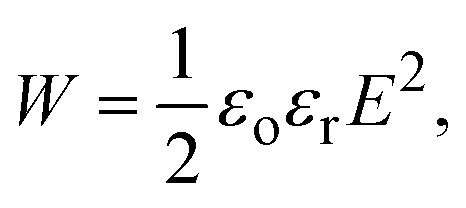
where *E* is the electric field, *ε*_o_ and *ε*_r_ are the free space permittivity and relative dielectric permittivity, respectively. However, nonlinear perovskites exhibit some energy losses, which affect the energy storage density. Their performance is determined in terms of recoverable energy storage (*W*_rec_) and energy storage efficiency (*η*) as follows:3
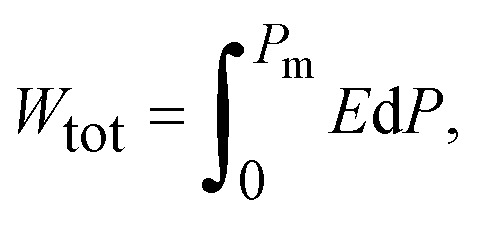
4
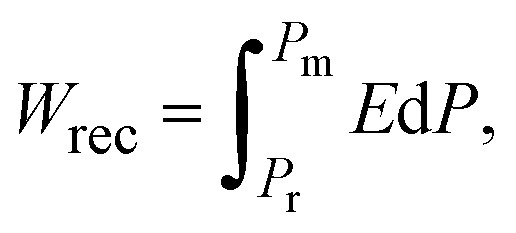
5
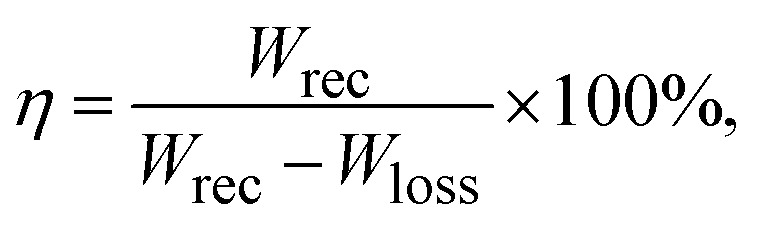
where *E* is the external electric field, *P* is polarization, *P*_m_ is maximum polarization, *P*_r_ is remanent polarization, and *W*_loss_ is energy loss, as displayed in Fig. 10(c). Notably, eqn (4) and (5), as well as Fig. 12(c), show that recoverable energy and efficiency are the most important parameters for evaluating the energy storage performance of Si-doped CsSrI_3_. It can be seen that high *ε*_r_ and maximum polarization are key to achieving higher energy density, while low dielectric and low remanent polarization improve energy storage efficiency. Table 3 displays some dielectric constants of some energy storage materials based on previous works.

**Table 3:** Reviewed dielectric properties of some energy storage materials

Works by:	Band gap (eV)	Dielectric constant	Ref.
Huang & Lambrecht (2016)	3.8–4.2	∼85	114
Wang *et al.* (2022)	3.9	∼90	115
Don *et al.* (2021)	3.8	∼95	
Miah *et al.* (2024)	3.7	∼100	116
Ravichandran *et al.* (2016)	3.9	∼88	117
Lee *et al.* (2018)	3.8	∼92	118
Noda *et al.* (2020)	3.85	∼90	119
Aspnes *et al.* (1984)	3.8	∼85	120
Mijalkovic (1995)	3.9	∼87	
Zeidi *et al.* (2023)	3.7	∼93	

### 3.2 CsSrI_3_ battery and capacitor integration

**Fig. 13:**
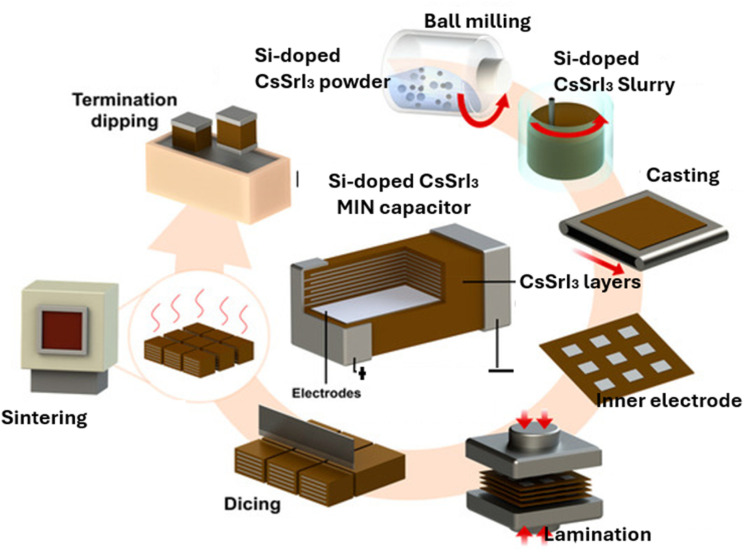
Schematic of the Si-doped CsSrI_3_ fabrication process according to Srinivas *et al.*^123^

Besides energy generation, CsSrI_3_ perovskites have been employed as scintillators and projected as future materials for solar cell applications. Their high ionic conductivity makes them strong materials for use as electrolytes, particularly in lithium-ion energy systems. According to Sonia *et al.*, incorporating Si-doped CsSrI_3_ into lithium-ion energy storage systems could significantly enhance energy density and charge/discharge rates.^121^ They also reported that ABX_3_ are high-conductivity materials that are utilizable as supercapacitors, requiring the fast transport of ions to attain power densities and rapid charge/discharge rates. Recently, Srinivas *et al.* (see Fig. 13) conducted a review on multilayer ceramic capacitors (MLCCs) such as CsSnI_3_, CsPbI_3_ and CsSrI_3_, and outlined various stages of their fabrication processes.^122^ According to them, the process involved various stages, such as ball milling, slurry formation, tape casting, screen printing, stacking/lamination, dicing, sintering, and termination dipping. The current review also adopted Srinivas *et al.*'s^123^ approaches to design fabrication process of Si-based CsSrI_3_ MIM capacitors, as shown in Fig. 13.

## 4 DFT-based approach and analysis

**Fig. 14:**
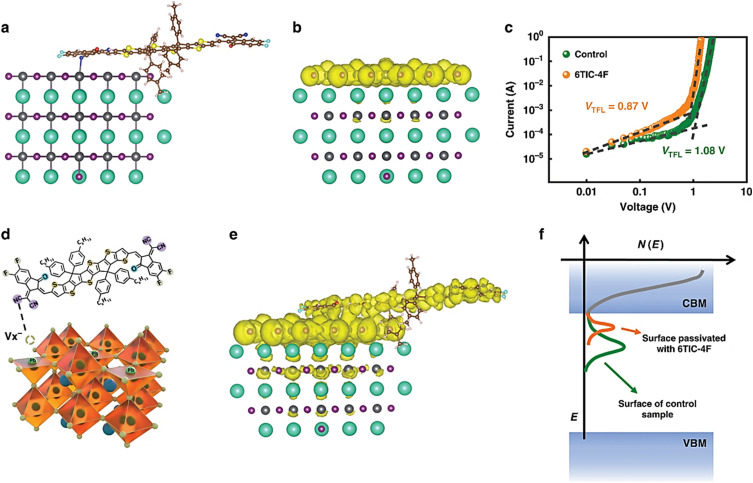
Schematic DFT profile of the perovskite and 6TIC-4F structures. Copyright@CC BY. (a) DFT-optimized interface between CsSrI_3_ perovskite and 6TIC-4F molecule. (b) Schematic surface adsorption of 6TIC-4F on perovskite layer. (c) Current–voltage characteristics showing reduced trap-filled limit voltage with 6TIC-4F. (d) Defect passivation mechanism of 6TIC-4F at vacancy sites in CsSrI_3_. (e) Surface-covered perovskite illustrating improved interface uniformity. (f) Density of states showing defect passivation and reduced trap states near band edges.

Jing *et al.* (see Fig. 14(a–f)) conducted a DFT-based experiment on CsPbI_*x*_Br_3−*x*_ capacitors based on surface passivation effects.^124^ The procedure and optimization process of their model are shown in Fig. 14, achieving a formation energy of −3.14 eV. They also identified a Boltzmann distribution ratio exceeding 99%, which is likely to be the most effective electron-donating group to passivate most of the surface traps. Their simulation was found to be consistent with XPS data in the interaction between Pb and CN/-S. However, removing one electron causes electron density localization on the Pb exposed surface, typically indicating surface traps (Fig. 14(b)). In Fig. 14(e), the valence electron densities shift toward some clusters, indicating the elimination of surface traps after passivation.

Due to limited data on previous works done on CsSrI_3_, we conducted a few DFT simulations on the electronic and dielectric properties of Si-doped CsSrI_3_ and analysed the results obtained. Within the limits of DFT and Quantum ESPRESSO, a fully optimized geometrical system of pristine CsSrI_3_ was generated using the BURAI optimizing tool. The system was appropriately relaxed till the individual atomic force was less than 0.02 eV per atom. The Si-doped perovskite variant was generated through the substitutional replacement of the Sr atom. To obtain the ground-state properties, the generalized gradient approximation (GGA) method was employed, using the Perdew–Burke–Ernzerhof (PBE) exchange correlation functional. The GGA-PBE was used because it performs well for periodic systems and minimizes overlap-related errors. The convergence criterion was achieved by successive *e*_cut_ and *k*-point convergence tests, which settled at 60 Ry and 7 × 7 × 7 *k*-grids, respectively.

**Fig. 15:**
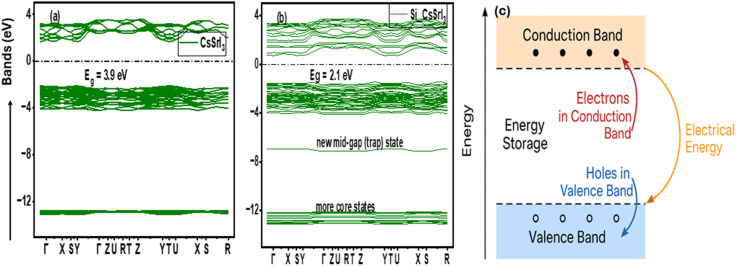
DFT-simulated electronic band structures for (a) pristine and (b) Si-doped CsSrI_3_. (c) Overall energy storage mechanism of the systems.

**Fig. 16:**
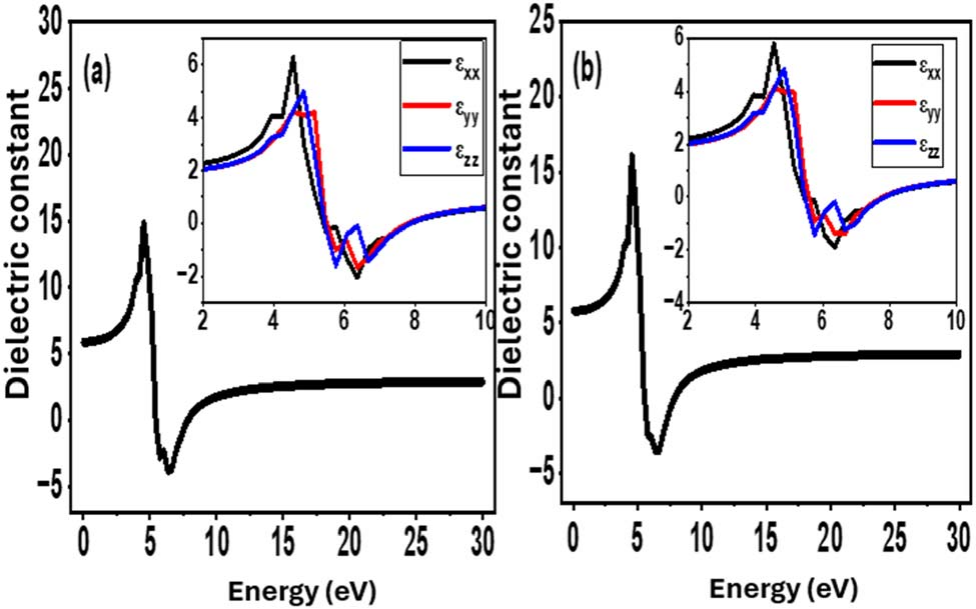
Dielectric profiles for (a) pristine and (b) Si-doped CsSrI_3_.

In Fig. 15(a), the band structure of pure CsSrI_3_ showed significant widening (3.9 eV), aligning with the reported range of 3.8–4.0 eV for halide perovskites, typically indicating highly insulating properties.^125^ Due to Si-dopants (Fig. 15(b)), significant tuning to 2.1 eV was obtained, coupled with the formation of core valence band states and other new states at the conduction band minimum (CBM). The new states at the CBM form a trap state, holding excess electrons for controlled discharge whenever the circuit needs them. Additionally, a moderate band gap value of 2.1 eV aligns with the reported value necessary to mediate a balance between excess leakage and performance,^126^ since the CB consists of virtually empty energy states and is ready to accept more electrons. Applying an external field enables the electrons in the VB to gain sufficient energy to move across the Fermi level to the CB. Because of this, more charges accumulate in the CB, making it store more electrical energy as they are at a higher energy state compared to their original position in the valence band. The resulting energy is stored as an electric field due to leakage prevention by the dielectric material. The overall mechanism of the energy storage process is illustrated in Fig. 15(c). As voltage is applied, electrons in the VB become excited, leaving behind holes because the electrical energy input drives electrons upward from the valence to the conduction band. They are prevented from drifting back to the VB by a trap layer (mid-gap state) formed by participating orbitals in the Si-dopant. In the dielectric constant *vs.* energy diagram presented in Fig. 16(a and b), both pure and Si-doped CsSrI_3_ show an improved static dielectric constant value of 17, which is well above the minimum range of 15 for perovskites. Si-atoms significantly increase static dielectric constant from 15.5 to ∼17. In the low (static) region, the dielectric constant dominates charge storage, whereas higher region peaks represent the electrical polarization necessary for improved charge storage. According to Abdu *et al.*, a high dielectric constant provides enhanced dielectric efficiency and reduces energy dissipation in PVA/PVP blend films boosted by MWCNTs/AuNPs.^127^ Nazia *et al.* obtained a band gap value of 2.9 eV in their research on engineering the band gap of double perovskites Na_2_LiYX_6_ (X = Cl and Br) using halide ion substitution, for energy harvesting.^128^ Notably, they found these perovskites to be highly suitable for energy storage applications due to a tunable band gap, enhanced dielectric constant (>15), reduced leakage and better structural stability.

Within the concept of Born effective charges (BEC), we also quantified the atomic displacements-induced polarization in Si-doped CsSrI_3_, for conversion from dynamic to electronic properties.^129^ According to Eric (2023), a proper understanding of BEC in perovskites highlights predictive ferroelectricity, capacitance and tunability for designing high-performance capacitors and energy storage systems.^130^ Herein, the computational details for determining BEC for CsSrI_3_ have been designed based on Koji *et al.*^131^6
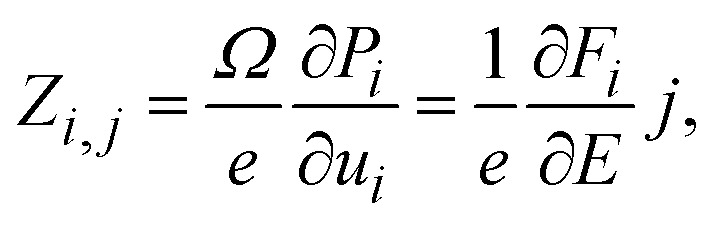
where *Ω* and *e* are the cell volume and the elementary charge, respectively; *P*_*i*_ and *u*_*j*_ are the macroscopic polarisation and atomic coordinates, respectively; *F*_*i*_ and *E*_*j*_ are the atomic forces and the electric field, respectively; subscripts *i* and *j* represent the *x*, *y*, or *z* directions.

**Fig. 17:**
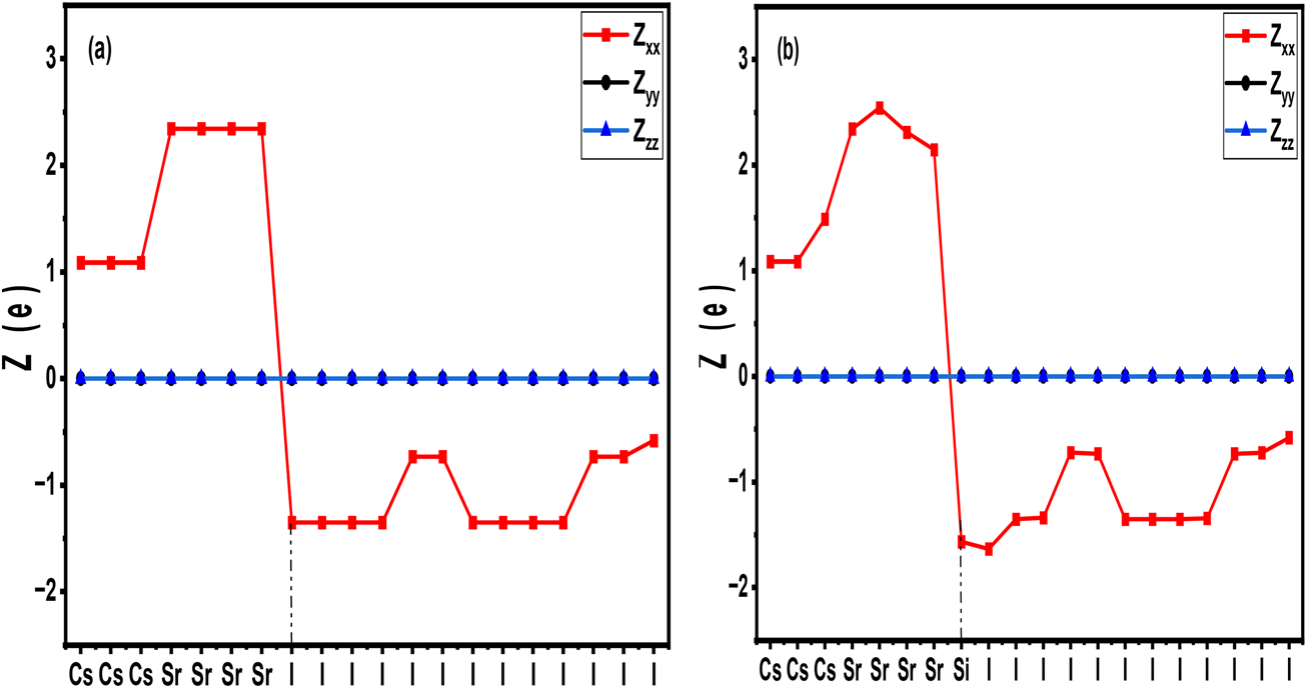
BEC profiles of the CsSrI_3_ dielectric materials: (a) pure CsSrI_3_ and (b) Si-doped variant.

Several BEC analyses revealed microscopic atomic properties with macroscopic charge storage efficiency.^132–134^ Based on the BEC graphs in Fig. 17, there is an enhanced strong interplay between atomic motion and polarization, and this has a direct influence on the dielectric properties and charge storage mechanisms. The BEC profile of pure CsSrI_3_ (Fig. 17(a)) shows a significant variation in *Z*_*xx*_, with the Cs ion displaying positive charge domination (1.5–2 e), which leads to strong polarization. The largest positive value is attributed to Sr (3 e), in good agreement with Nocole *et al.*'s work on the charge analysis of the SrTiO_3_ perovskite, while negatively charged I ions (−1 e) balance the overall polarization. With Si dopants (Fig. 17(b)), Cs and Sr maintained similar trends to the pristine lattice, with a slight distortion near Sr induced by the Si atom, which reduced symmetry. Contrary to the higher variation induced by Cs, I atoms exhibited a subtle shift, implying a modified bonding environment. Upon general observation, Si doping slightly improves the Cs contribution and perturbs the Sr–I interaction, potentially increasing anisotropy and enhancing charge storage capacity through the improved polarization tunability.

### 4.1 Processing and compatibility with MIM architecture

**Fig. 18:**
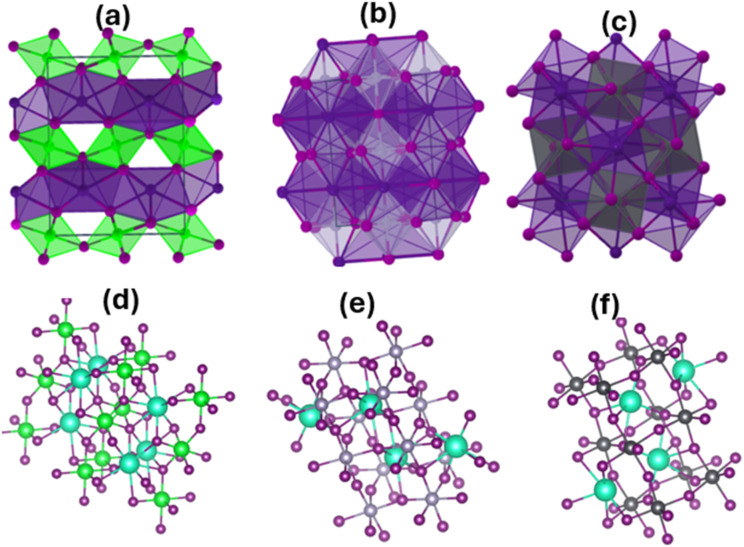
Crystal frameworks of the halide perovskites (a) CsSrI_3_, (b) CsSnI_3_ and (c) CsPbI_3_. (d–f) Atomic clusters of the corresponding crystal frameworks. Source: open source from the Materials Project.

CsSrI_3_ is a halide perovskite, similar to CsSnI_3_ and CsSnBr_3_,^135^ which crystallizes in the orthorhombic *Cmcm* space group. The crystal frameworks (Fig. 18(a–c)) and the atomic clusters (Fig. 18(c–e)) of the perovskites CsSrI_3_, CsSnI_3_, and CsPbI_3_ are displayed. The atomic clusters show Cs (large spheres), B-site cations (Sr, Sn, Pb), and I atoms (smaller spheres). CsSrI_3_ exhibits a more open framework compared to the dense connectivity in CsSnI_3_ and CsPbI_3_. The corner-sharing SrI_6_ octahedra exhibit 8–12-fold coordination of Cs atoms. Based on DFT data, the halide perovskite band gap ranges between 3.8 and 4 eV, with a moderate static dielectric constant of 6.5.^136,137^ These properties indicate an intrinsic insulating behaviour, which is efficient for an MIM stack. From a processing point of view, CsSrI_3_ (and other CsSr/SnX_3_ halide perovskites) can form thin films *via* vacuum co-sublimation or solution routes.^138^ Based on previous works on similar Pb systems, vacuum deposition and two-step conversions demonstrate controllable crystallization, surface morphology, and leakage suppression—principles transferable to CsSrI_3_ with careful moisture/oxygen exclusion and iodine stoichiometry control.^139^ Kushagra *et al.* reported that halide perovskites (CsSnI_3_, CsPbI_3_, CsPbBr_3_, *etc.*) are highly recognized as high-k dielectric materials in MIM-type capacitors and optoelectronics, with an emphasis on band-offset engineering and leakage regulation.^140^ Halides with a wide gap use electrodes with maximum barrier heights, such as TiN, to ensure smooth, pinhole-free films. Table 4 reveals some comparative results based on the effects of dopants in regulating the key energy storage properties of perovskites. Across the perovskite systems listed in Table 4, introducing dopants regulates band gap, dielectric constant, and band alignment. Several dopants, such as Li in CsPbI_3_ (*E*_g_ = 1.65 eV, *ε*_r_ = 25) and Cu in Cs_2_AgBiBr_6_ (*E*_g_ = 1.98 eV, *ε*_r_ = 12), show significant band gap narrowing, enhancing carrier polarizability with an increased dielectric response. However, their relatively small gaps can increase the risk of leakage under high electric fields. Similarly, K-doped CsPbBr_3_ (*E*_g_ = 2.3 eV, *ε*_r_ = 18) and Mn-doped Cs_2_AgBiBr_6_ (*E*_g_ = 1.9 eV, *ε*_r_ = 12) show moderate band-gap narrowing coupled with defect-related mid-gap states, which compromise the insulating behaviour. Within this landscape, based on the compatibility of properties obtained from other halide perovskites (Table 4), the high dielectric constant of orthorhombic Si-doped CsSrI_3_ (>15) indicates moderate capacitance density. Therefore, doping with Si will enhance the BEC, with a significantly higher density, while keeping the breakdown strength acceptable. Moreover, most of the perovskites show a band gap between 0.55 and 3.6 eV. The narrow gap perovskites, such as Cs_2_AgRhCl_6_, are at a high risk of leakage, while the widest-gap perovskite, like CsSnCl_6_, shows a reduced dielectric response. Si-doped CsSrI_3_ showed a band gap of ∼2.1 eV, which is ideal. It is wide enough to suppress leakage and efficient for strong polarization and BEC coupling.^141^ Notably, the Si-doped CsSrI_3_ positions itself between CsPbBr_3_ (2.3 eV) and Cs_2_AgBiBr_6_ (1.9 eV), which are both considered good candidates for capacitors. In terms of the dielectric constant, CsPbI_3_ (Li-doped) takes the lead, while Si-doped CsSrI_3_ shows a value that is significantly higher than most of the double-halide perovskites. Its stable band alignment is also compatible with the TiN/Pt electrodes in MIM stacks. Additionally, Si-doped CsSrI_3_ can be considered Pb-free and environmentally safer than CsPb-based systems.

**Table 4:** Some halide perovskites doped for energy storage

Perovskite	Dopant	Band gap (eV)	Dielectric constant	Band offset	Ref.
CsPbI_3_	Li	1.65	25	Type-I alignment with TiN	142
Cs_2_AgBiBr_6_	Cu	1.98	12	Indirect band gap with lowered conduction band	143
CsPbBr_3_	K	2.3	18	Downshifted VB by 0.2 eV	144
Cs_2_SnCl_6_	Vacancy type	3.6	10	Wide gap, low dielectric	145
Cs_2_AgBiCl_6_	Bi	2.2	11	Type II alignment with Au	146
Cs_2_AgSbI_6_	Rb	0.94	15	Narrow with offset of 0.3 eV	147
Cs_2_AgBX_6_	X = Br, I, and Sb	1.0–1.5	13	Tunable halide-based band offset	148
Cs_2_AgRhCl_6_	Alkali	0.65	14	Direct gap with strong Rh–Cl hybridization	149
Cs_2_AgBiBr_6_	Mn	1.9	12	Mid-gap formed due to Mn	150
Cs_2_RhAgX_6_	Cl, Br, and Ir	0.55–2.2	16	Indirect gap with strong spin–orbit coupling	
CsSrI_3_	Si	2.1	16.6	Direct gap with mid-gap state by Si orbitals	150

### 4.2 Reliability under thermal and electrical stress

The thermodynamic resilience of halide perovskites is critical for their operation in high-temperature conditions.^151^ Reports have indicated that doped CsSrI_3_ shows strong stability under both thermal and electrical stress, making it a good choice for energy storage applications.^152^ For example, the orthorhombic structure of the Si-doped CsSrI_3_ lattice creates a stable Si–I covalent bond network that resists thermal distortion.^153^ While other perovskites like CsPbI_3_ suffer from phase transition problems and easy decomposition above 150 °C, the 250 °C synthesis of Pr^3+^-doped CsSrI_3_ by Qian *et al.* showed that this material can maintain structural integrity at high temperatures because of the Sr–I framework and reduced ionic migration pathways.^154^ The 2.1 eV band gap under Si-doping is enough to prevent leakage under a high external field strength, better than narrow-gap perovskites like Cs_2_AgRhCl_6_. Therefore, Si-doped CsSrI_3_ can perform with moderate conduction and controlled voltages. The DFT-obtained BEC and the enhanced dielectric constant show strong polarization without compromising insulation. The localized mid-gap states formed in the Si-doped materials do not allow conductive channels, which preserves the breakdown strength better than CsPbBr_3_-based dielectrics. The review by Sile *et al.* covered the potential of metal halide perovskites as emerging thermoelectric materials.^155^ They identified ultra thermal conductivity coupled with a high Seebeck coefficient as driving forces for their thermoelectric behaviours. Investigations of the thermal properties of metal halide perovskites began in the early 1970's, which paved the way for a better understanding of their structural phase change. Subsequently, thermal conductivity analysis has been carried out on most halide perovskites, and it has been resolved that thermal transport is reduced for crystal grains with dimensions smaller than 100 nm because photons have mean free paths of the same order. Higher thermal conductivities, up to 0.46 W (mK)^−1^, have been reported for CsPbBr_3_, which belongs to the same family as CsSri_3_.^156^ Tobias *et al.* also reported that the thermal conductivity is lower near grain boundaries. The findings from these studies revealed that heat evolution in dynamically operated halide perovskites is inefficient, thereby limiting their optoelectronic applications.^156^

## 5 Conclusions and perspectives

The intrinsic instability of halide perovskites in the presence of moisture, oxygen, and thermal stress remains a key challenge. CsSrI_3_ may undergo structural degradation or halide migration, which can affect dielectric performance and long-term operations. Although using Si may enhance chemical robustness, the process requires careful experimental and theoretical synergy regarding solubility limit and structural distortions. Charge compensation *via* iodine vacancy can lead to shallow defect levels, facilitating leakage in MIM configurations. It is also necessary to add optimal doping concentration to prevent defect suppression by Si impurities. CsSrI_3_ must also withstand standard fabrication temperatures, interface cleanly with metal electrodes, and avoid diffusion or phase transitions during device operation.

The wide band gap of CsSrI_3_ offers exciting potential, which paves the way for Si incorporation to increase breakdown voltage and reduce leakage currents. The tunable dielectric constant of the Si-doped material enables optimization for high-density MIM capacitors. The current review found that CsSrI_3_ and other halide perovskites have relatively low production cost and are solution-processable. This suggests a pathway for fabricating flexible MIM capacitors with large surface areas. With careful stability control, Si-doped CsSrI_3_-based dielectrics could outperform conventional oxides such as SiO_2_, Al_2_O_3_, or HfO_2_ in specific high-capacitance applications. From the experimental perspective, it is necessary to optimize low-temperature deposition pathways and interface with materials such as Au, Pt, or TiN for improved reliability. The strategic determination of the frequency-dependent dielectric response, breakdown behaviour, and endurance cycling would further aid the feasibility of Si-prompted CsSrI_3_ in real-world MIM capacitor architectures.

Future research shall pay attention to defect passivation strategies, such as co-doping, halide supplementation, or surface functionalization to suppress vacancies and ion migration. Advanced DFT studies on the effects of Si on the electronic structure, phonon behaviour, and dielectric tensor will be essential for guiding synthesis.

Controlling the preparation of Si-doped CsSrI_3_ perovskite materials for the dominant Si-based capacitors is a crucial step to realizing practical perovskite-based MIM devices. The current review has identified CsSrI_3_ as a member of the halide perovskite family with a tunable band gap for energy storage applications. Although its development is still in the early stages, the review has identified several scientific and engineering challenges that must be addressed before it can be fully adopted in microelectronic capacitor architectures. Recently, the performance and technology of optoelectronic devices based on the integration of halide perovskites modified with Si-based materials have substantially improved. In the context of battery and capacitor integration, Si-doped CsSrI_3_ has high ionic conductivity, which positions it as an emerging electrolyte that is efficient for MIM capacitor integration. The key challenge regarding its fabrication is iodine loss, which becomes severe above 150–250 °C. Based on the simulation results, Si-doping in CsSrI_3_ induces the formation of core valence band states and other new states at the conduction band minimum, which holds electrons for controlled charge/discharge. The dielectric profiles were also found to align with the other halide perovskite MIM architectures. The BEC approach shows that Si doping slightly improves Cs contribution and perturbs Sr–I interactions, potentially increasing anisotropy and enhancing the charge storage capacity through improved polarization tunability. Si-doped CsSrI_3_ and related halide perovskites offer low-cost, solution-processable routes for flexible, large-area MIM capacitors. With improved stability control, these dielectrics could surpass conventional oxides in high-capacitance applications. Experimentally, optimizing low-temperature deposition and stable interfaces with materials like Au, Pt, and TiN is essential for reliable device performance. Detailed studies of frequency-dependent dielectric behavior, breakdown strength, and cycling endurance will determine practical viability. Future research should focus on defect passivation—through co-doping, halide supplementation, and surface functionalization—and use advanced DFT to understand how Si affects the electronic structure, phonons, and dielectric properties. Si-doped CsSrI_3_ offers an environmentally sustainable alternative to lead-based perovskites for energy storage. Silicon doping enhances the structural stability, prolonging material lifespan and reducing waste. Its non-toxic, lead-free, and abundant composition supports renewable energy integration, minimizes ecological hazards, and aligns with global efforts toward green technologies and resource-efficient energy systems.

## Conflicts of interest

The authors declare no known conflicts of interest.

## Data Availability

The code for Quantum ESPRESSO used to generate new data for this review can be found at https://www.quantum-espresso.org/. The version of the code employed for this study is version 7.4.
